# Decaying Post-Seismic Deformation Observed on the Korean Peninsula Following the 2011 Tohoku-Oki Earthquake

**DOI:** 10.3390/s21134493

**Published:** 2021-06-30

**Authors:** Dong-Hyo Sohn, Byung-Kyu Choi, Sungshil Kim, Sun-Cheon Park, Won-Jin Lee, Pil-Ho Park

**Affiliations:** 1 Space Science Division, Korea Astronomy and Space Science Institute, 776, Daedeokdae-ro, Yuseong-gu, Daejeon 34055, Korea; bkchoi@kasi.re.kr (B.-K.C.); phpark@kasi.re.kr (P.-H.P.); 2 Department of Earth Science Education, Chonnam National University, 77, Yongbong-ro, Buk-gu, Gwangju 61186, Korea; neogeo94@jnu.ac.kr; 3 Earthquake and Volcano Research Division, Korea Meteorological Administration, 61, Yeouidaebang-ro 16-gil, Dongjak-gu, Seoul 07062, Korea; suncheon@kma.go.kr; 4 Environmental Satellite Center, National Institute of Environmental Research, 42, Hwangyong-ro, Seo-gu, Incheon 22689, Korea; wjleeleo@korea.kr

**Keywords:** GNSS, 2011 Tohoku-Oki earthquake, Korean Peninsula, crustal movement, recovery patterns

## Abstract

We investigated decaying post-seismic deformation observed on the Korean Peninsula associated with the 2011 Mw 9.0 Tohoku-Oki earthquake using Global Navigation Satellite System (GNSS). The GNSS velocity vectors were estimated in five periods from 2005 to 2019. A co-seismic offset of the Korean Peninsula caused by the 2011 earthquake was inversely proportional to epicentral distances. According to the temporal variations of two components (magnitude and direction) of the GNSS velocity vector with the epicentral distance, the difference between the eastern and western regions for the two components becomes smaller over time. For approximately nine years after the 2011 event, the direction for the crustal movement in South Korea showed a recovery pattern returning to the pre-earthquake motion. In addition, the recovery patterns of the crustal movement were observed differently with the regional geologic structure (e.g., the crustal thickness) and each period. Our estimates of the decay in post-seismic deformation of the Korean Peninsula suggest that post-seismic relaxation will be complete within 5–20 years after the 2011 earthquake. The results suggest that the crustal movement on the Korean Peninsula is gradually recovering to its pre-earthquake motion.

## 1. Introduction

Many earthquakes occur somewhere on Earth every day. Most earthquakes are so weak that they rarely cause ground displacements, but some large earthquakes produce significant crustal deformation over large distances. Studies of major earthquakes with magnitudes exceeding 7.0 play a key role in improved understanding of crustal deformation, including co-seismic and post-seismic effects [1]. Many studies have been conducted on major earthquakes to investigate earthquake-induced crustal deformation and evaluate the geodynamic effects [2,3,4]. In particular, megathrust earthquakes create not only considerable co-seismic displacements, but also post-seismic deformation over a long duration.

The Mw 9.0 Tohoku-Oki earthquake, which struck northeastern Japan on 11 March 2011, is one of the most severe natural disasters ever recorded. It represents the fourth-largest earthquake to occur since 1900 [5,6]. Ozawa et al. [4] reported that the onshore co-seismic displacement nearest to the epicenter of the Tohoku-Oki earthquake reached approximately 5.3 m. In addition, Tobita [7] suggested that there has been a continuous increase in the contribution of viscoelastic relaxation to post-seismic deformation in eastern Japan, whereas the contribution of afterslip has rapidly decreased using combined logarithmic and exponential decay function model. Other studies found that the Pacific coastal area near the epicenter of the Tohoku-Oki earthquake in a latitude range between 36° and 39° was uplifted due to viscoelastic relaxation [8,9].

**Figure 1:**
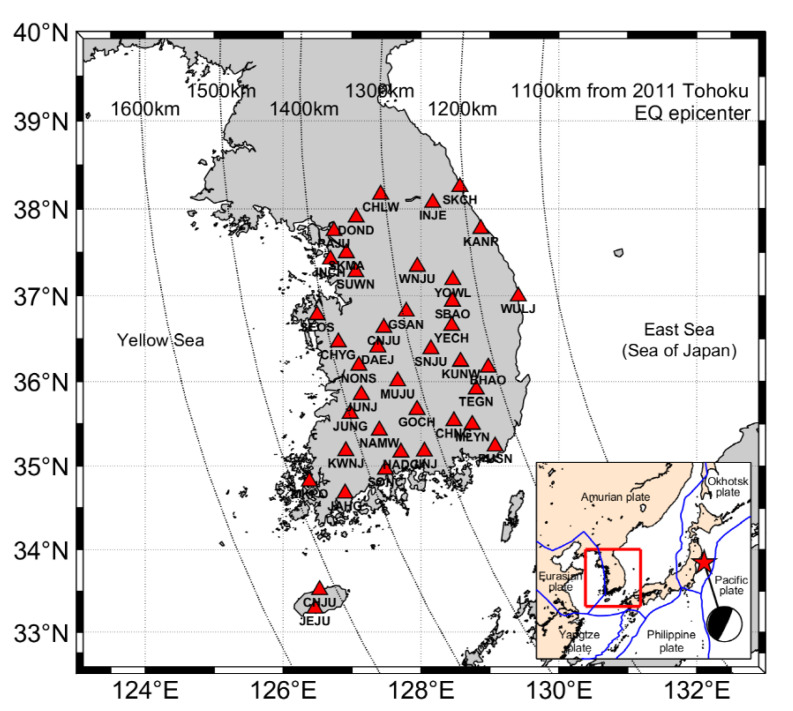
Locations of continuously operating GNSS stations (red triangles). The curved dotted lines represent the distance from the epicenter. Inset: The star marks the epicenter of the 2011 Tohoku-Oki earthquake. The blue lines represent the plate boundaries.

The Korean Peninsula is located near the margin of the Amurian microplate (Figure 1). It is tectonically stable compared with other regions in East Asia [10,11]. Some studies have shown that the crust of the Korean Peninsula moves to the southeast at approximately 30 mm/year on average in the International Terrestrial Reference Frame (ITRF) 2000 frame [12,13]. No significant earthquake with a magnitude of over 6.0 has ever been recorded in the Korean Peninsula [14]. However, the Korean Peninsula can be indirectly affected by a major earthquake occurring in neighboring Japan.

Previous studies have reported that the 2011 Tohoku-Oki earthquake strongly affected the crust of the Korean Peninsula [15,16,17]. According to the results of their investigations, an average co-seismic deformation of approximately 30 mm was observed, although the Korean Peninsula is more than 1000 km away from the epicenter. This amount is similar to the crustal movement of the Korean Peninsula for one year. In particular, the eastern coastal region of the Korean Peninsula closest to the epicenter moved approximately 40 mm toward the epicenter due to the 2011 Tohoku-Oki earthquake, while the far western coastal region was observed to exhibit an average displacement of approximately 10 mm [18,19]. This displacement decreased with an increase in epicentral distance.

Kim et al. [20] suggested that the 2011 Tohoku-Oki earthquake effects on the Korean Peninsula crustal deformation lasted for approximately two years. However, they analyzed it based on changes in the average velocity with an interval of two years for only about six years after the mainshock. On the other hand, Kim et al. [21] suggested that post-seismic deformation for about 2.7 years after the 2011 earthquake is approximately 80% complete using GPS data from 12 March 2008 to 30 November 2013. Completeness is based on analyzing the exponential function curve fitting of the post-seismic deformation time series. It is defined as the ratio of the cumulative post-seismic displacement for approximately 2.7 years to the final estimated cumulative displacement. In contrast, Shao et al. [17] calculated the theoretical post-seismic crustal deformation based on a viscoelastic medium model. They proposed that although the post-seismic crustal deformation decays exponentially with time as a whole, a linear variation could last for 200 years following the Mw 9.0 Tohoku-Oki earthquake at a station in China located 1276 km away from the epicenter.

In this study, we calculate crustal velocity vectors in five periods (2005–2010, 2011 co-seismic, 12 March 2011 to 2013, 2014–2016, 2017–2019) for 15 years from 2005 to 2019. In addition, we investigate the crustal movement recovery in post-seismic deformation of the Korean Peninsula following the 2011 Tohoku-Oki earthquake. More specifically, we process data obtained from a global navigation satellite system (GNSS) network in the Korean Peninsula to investigate the pre-, co-, and post-seismic deformation. As mentioned earlier, there was no significant crustal deformation on the Korean Peninsula before the 2011 earthquake. Therefore, we analyze the relative changes of the velocity vector after the Tohoku-Oki earthquake by referring to the pre-seismic motion. The velocity vector can be separated into a magnitude and a direction. As a co-seismic offset of the Korean Peninsula caused by the 2011 earthquake is inversely proportional to epicentral distances, we analyze the temporal variations of the two components, magnitude and a direction of a velocity vector, with the epicentral distance. In addition, the recovery patterns of the crustal movement were investigated with the regional geologic structure. We also estimate the post-seismic relaxation completeness using an exponential decay function.

## 2. Data and Methodology

### 2.1. GNSS Data

GNSS is an effective geodetic tool for investigating the movement of the crust. We use displacements captured by GNSS stations within a network operated by the National Geographic Information Institute of Korea (NGII) and Korea Astronomy and Space Science Institute (KASI); accordingly, we determine the crustal motion of the Korean Peninsula. Figure 1 shows the geographic locations of the NGII and KASI GNSS stations employed in this study; the curved dotted lines represent the distance from the 2011 Tohoku-Oki earthquake epicenter. We analyze 40 GNSS stations on the Korean Peninsula for 15 years from 2005 to 2019.

### 2.2. Methodology

GNSS data with a sampling interval of 30s were processed in the International GNSS Service (IGS) realization of the reference frame ITRF 2014 using a double-difference method in the Bernese 5.2 software [22]. In this study, we have used some IGS stations around the world as fiducial sites when processing the GNSS data. In addition, we use various additional products and models, such as precise GNSS orbits and clocks from the IGS, Earth rotation parameters, phase center offsets and variations for satellites and ground antennas, and tidal loading effects. Station solutions are eventually estimated in a geocentric Earth-fixed reference system composed of X, Y, and Z axes. Additionally, then, they are transformed to the topocentric coordinates according to the north, east, and up components. The supporting information Figure S1 is an example of converting geocentric coordinates (X, Y, Z) to topocentric coordinates (N, E, V).

We processed the data to determine average velocity vectors for the GNSS stations over each of five periods; i.e., pre-seismic (1 January 2005 to 31 December 2010), co-seismic (4 March 2011 to 18 March 2011), post-seismic stage 1 (12 March 2011 to 31 December 2013), post-seismic stage 2 (1 January 2014 to 31 December 2016), and post-seismic stage 3 (1 January 2017 to 31 December 2019). In the Korean Peninsula, there have no records of earthquakes of magnitude 6 or greater, and only two earthquakes of magnitude 5 or greater in the 30 years before the 2011 earthquake [14]. Additionally, it has an average of about 30 mm movement per year without any significant crustal deformations [12,13].

Some studies suggested that the earthquake-induced significant effects lasted for 2~2.5 years after the 2011 mainshock [7,9,20]. However, Blewitt and Lavallée [23] recommended that a minimum time span of 2.5 years be adopted for velocity solutions intended for tectonic interpretation or reference frame production. It is to minimize the effect of seasonal noise in the GNSS data on the fitting. Therefore, we divided the period into intervals of about 3 years after the 2011 earthquake.

We consider the linear regression fitting approach to estimate the velocity vectors during each time period. In this study, we use a simple linear regression model that describes the relationship between a dependent variable *Y* (e.g., north, east, or up components values) as a function of one independent variable *X* (e.g., observation time). The general equation for a simple linear regression can be expressed by the following Equation (1). This model can also be represented in matrix form as Equation (2).

(1) Y=α+βX+ε

(2) $$\left[\begin{array}{c} Y_{1}\\ Y_{2}\\ \vdots \\ Y_{n} \end{array}\right]=\left[\begin{array}{cc} 1 & X_{1}\\ 1 & X_{2}\\ \vdots & \vdots \\ 1 & X_{n} \end{array}\right]\left[\begin{array}{c} \alpha \\ \beta \end{array}\right]$$

where *α*, *β*, and *ε* are the intercept, slope, and error, respectively. The coefficient confidence intervals for the slope are calculated using the Wald method expressed in Equation (3) [24].

(3) $$\hat{\beta }\pm t_{\left(1-\alpha /2,n-p\right)}se\left(\hat{\beta }\right)$$

where $\hat{\beta }$ is an estimate of coefficient, $se\left(\hat{\beta }\right)$ is the standard error of the coefficient estimate, and $t_{\left(1-\alpha /2,n-p\right)}$ is the 100(1 − *α*/2) percentile of *t*-distribution with *n* − *p* degrees of freedom that leaves a probability *α*/2 in the upper tail and (1 − *α*/2) in the lower tail. *n* and *p* are the number of observations and the number of regression coefficients, respectively. Here, we use the 95% confidence intervals (i.e., α = 0.05). The supporting information Figure S2 shows examples of time series, annual velocities, and 95% confidence interval values of the north, east, and up components for some GNSS stations.

Only the horizontal components of velocity were used in the analysis of crustal motions caused by the earthquake; vertical displacements in the region were comparatively insignificant [25,26]. We separate the horizontal velocity vectors into their magnitude and direction components and analyze the temporal variations of these two components depending on the epicentral distance.

In addition, we investigate the post-seismic deformation associated with the 2011 Tohoku-Oki earthquake. Post-seismic deformation is observed in GNSS time series following a large earthquake. In general, post-seismic GNSS time series can be fitted by a logarithmic, exponential, or combined decay function [2,7,8]. In this study, we apply an exponential decay function that is a simple decay function to investigate post-seismic deformation [27,28]. Before modeling, we removed the secular motion of tectonic plates and the signal pertaining to the annual cycle from the coordinate time series [21]. By removing these effects, the total post-seismic displacements associated with the Tohoku-Oki earthquake could be accurately monitored.

## 3. Results

### 3.1. Changes in Average Velocity Vector

We investigated any changes in average velocities over five periods, as summarized in Table 1. Additionally, the velocity vectors of all stations for each period are summarized in Table S1 of the supplementary material. Before the 2011 Tohoku-Oki earthquake, the crust of the Korean Peninsula moved at an average rate of approximately 30 mm/year in the direction of 119 degrees. In the stage of co-seismic deformation, we pre-calculated the co-seismic offset that is the difference between an averaged position in the week before and the week after the earthquake. The predominant direction of co-seismic deformation is about 84 degrees with magnitudes of 23 mm on average. It is noted that the difference in the orientation of the pre-seismic and co-seismic vectors is more than 30 degrees, and the co-seismic displacement direction was rotated counterclockwise toward the epicenter. In the post-seismic stage 1 through 3, the average magnitude is about 36, 32, and 31 mm/year, respectively. The magnitude tends to return to the pre-seismic level gradually. Additionally, the average direction also shows a tendency to return to their pre-seismic condition by representing about 106, 111, and 115 degrees at each stage. In the post-seismic stage 3, the magnitudes are, on average, a few mm greater than the pre-seismic deformation. Additionally, the average direction differs about four degrees counterclockwise from the pre-seismic direction.

### 3.2. Co-Seismic Displacement with Epicentral Distance

The horizontal co-seismic displacements reached about 35 mm on the eastern coast of the Korean Peninsula, approximately 1150 km away from the epicenter. In contrast, the crustal displacement on the western coast area was approximately 10 mm on average. When the distance increases from the epicenter, the displacement amplitudes tended to decrease. The co-seismic movement for all stations is summarized in the supporting information Table S1. In addition, apparent crustal velocity differences were observed between the eastern and western regions of the Korean Peninsula. These results are consistent with results reported by Baek et al. [15], Ha et al. [16], and Shao et al. [17].

### 3.3. Variation of Velocity Magnitude with Epicentral Distance

**Figure 2:**
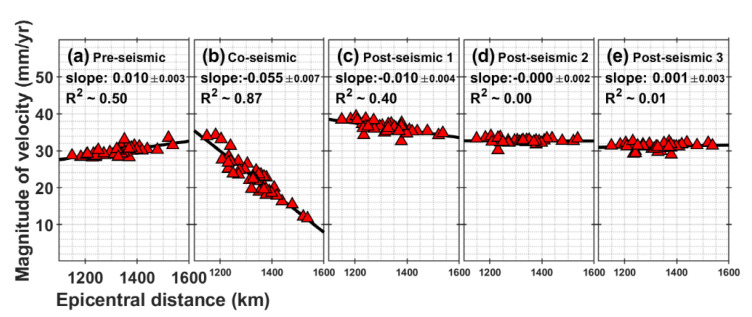
Magnitude of the horizontal GNSS velocity vectors with epicentral distance for each station in the five periods (i.e., (**a**) Pre-seismic, (**b**) Co-seismic, (**c**) Post-seismic 1, (**d**) Post-seismic 2, (**e**) Post-seismic 3) from 2005 to 2019. The red triangles represent the magnitudes of the velocity vectors for the GNSS stations in mm per year. The solid black lines (slope) are the best linear fits to the data. The coefficients and 95% confidence intervals for ‘slope’ are calculated as the linear relationship between two variables. R^2^ is the coefficient of determination for a best-fit line. Note: In the (**b**) co-seismic stage, the unit of magnitude is mm. It is a total displacement, not a rate.

As mentioned in the previous section, there was a difference of about 25 mm in co-seismic deformation between the east and west regions of the Korean Peninsula. Therefore, we analyzed the variation in the magnitude component of vectors over each of the five periods depending on the epicentral distance. Figure 2 shows the magnitudes of the horizontal GNSS velocity vectors as a function of the distance from the epicenter. In Figure 2, the slope represents the best linear fit to the data.

For the pre-seismic period, the best-fit line has a positive slope, which means that the crust of the western regions (far-field) moves a little more rapidly than that of the eastern regions (near-field) before 2011 (Figure 2a). In Figure 2b, however, we can observe that the Korean Peninsula experienced large co-seismic displacements due to the 2011 Tohoku-Oki earthquake. The displacement is approximately 34 mm within 1200 km from the earthquake epicenter, whereas the movement at distances > 1500 km is approximately 12 mm. There was a linear westward decrease. In the post-seismic stage 1, the velocity magnitudes in the eastern regions are still larger than those of the western regions (Figure 2c). However, in the post-seismic stage 2, the slope is zero (Figure 2d). This is indicating that the magnitude of the velocity in the eastern and western regions is similar. As shown in Figure 2c–e, it is remarkable that the slope changes gradually from negative to positive. In other words, a relation for the velocity magnitude between east and west regions tends to return to the pre-earthquake motion gradually. There was a slight difference in the magnitude between the eastern and western regions over time. In addition, R-squared (R^2^) values, the coefficient of determination for best-fit lines, become smaller. This means the weakness of the relationship between the epicentral distance and the magnitude over time. The overall magnitude of the velocity vector became more similar to that of the pre-earthquake. However, it has not fully returned, indicating that post-seismic deformation is ongoing.

### 3.4. Variation of Velocity Direction with Epicentral Distance

**Figure 3:**
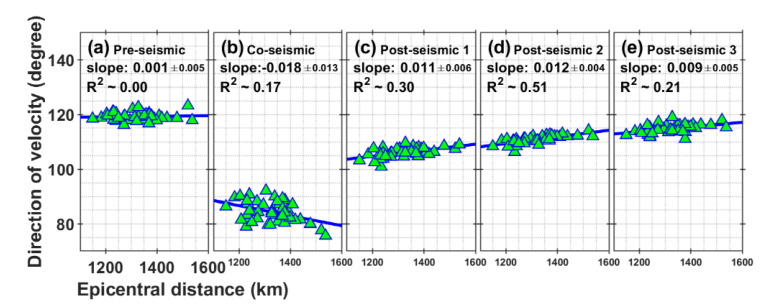
Direction of the GNSS velocity vectors with epicentral distance for each station in the five periods (i.e., (**a**) Pre-seismic, (**b**) Co-seismic, (**c**) Post-seismic 1, (**d**) Post-seismic 2, (**e**) Post-seismic 3). The direction of the velocity vector represents the angle in degrees clockwise from north (that is, north = 0 degrees and south = 180 degrees). The green triangles represent the directions of the crustal velocity vectors for the GNSS stations in degrees. The solid blue lines indicate the linear fit with the data. The coefficients and 95% confidence intervals for ‘slope’ are calculated as the linear relationship between two variables. R^2^ is the coefficient of determination for a best-fit line.

To investigate any changes in the direction of crustal movement between the eastern and western regions, we calculated the best-fitting slopes for the direction using two variables (the direction of the vector and the epicentral distance) in each stage during each time period. Figure 3 shows the direction of velocities for GNSS stations in five periods. As shown in Figure 3a, although the slope is positive and small, and there is a lot of scattering, the crust moves in a similar azimuth between the eastern and western regions for five years from 2005 to 2010. However, as shown in Figure 3b, the slope changes rapidly from positive to negative due to the 2011 megathrust earthquake, i.e., co-seismic displacement for the western sites is rotated counterclockwise relative to the eastern sites. However, it is a weaker relationship (R^2^ = 0.17) than the variation in velocity magnitude (R^2^ = 0.87; Figure 2b). In Figure 3c, the slope recovered to positive with a value of about 0.011. However, it differs from pre-earthquake with a value of 0.001. As shown in Figure 3c–e, the slope changed a small amount during these periods of post-seismic deformation. There is still a difference in the direction of movement between the east and west regions. It can be noted that the slope is very gradually getting closer to the pre-earthquake of Figure 3a.

### 3.5. Post-Seismic Deformation

To understand the displacement characteristics of the Korean Peninsula after the earthquake and to estimate the duration of post-seismic deformation, we applied a curve-fitting algorithm based on an exponential function using the GNSS time series. In addition, the time series datasets after the 2011 event are subtracted by the secular motion of tectonic plates. The applied exponential function for post-seismic deformation is given by Equation (4) [27,28].

(4) $$u\left(t\right)=c+a\left(1-e^{-\frac{t}{\tau }}\right)$$

In Equation (4), *u*(*t*) is the horizontal displacement of the GNSS station, *t* is the time after the 2011 earthquake, *c* is the co-seismic offset, *a* is the amplitude associated with the estimated final cumulative displacement, and *τ* denotes the decay time. From Equation (4), we compute the unknown parameters (*a*, *c*, *τ*) associated with the site displacements for the GNSS stations.

**Figure 4:**
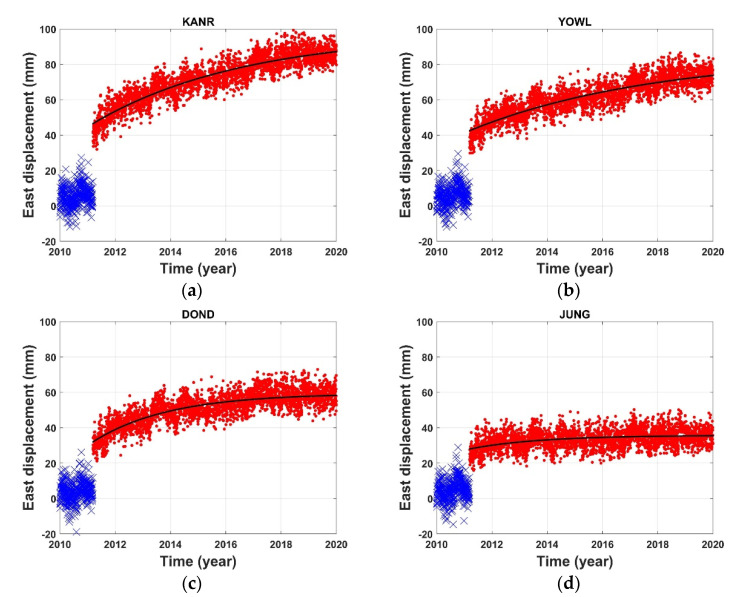
Post-seismic time-series for the east components of four GPS stations: (**a**) KANR, (**b**) YOWL, (**c**) DOND, and (**d**) JUNG. These four GPS stations are located 1183, 1228, 1339, and 1398 km away from the epicenter, respectively. The blue crosses represent the pre-seismic displacements, while the red dots denote the post-seismic displacements. The black lines are exponential fitted curves using GPS data.

Figure 4 shows the east–west post-seismic displacement time series for four GNSS stations (KANR, YOWL, DOND, and JUNG). These GNSS stations are approximately 1183, 1228, 1339, and 1398 km from the epicenter, respectively (KANR is the closest to the epicenter). As shown in Figure 4a, KANR exhibits a large co-seismic displacement of approximately 34 mm in the east–west direction during the 2011 earthquake; after the earthquake, the displacement has attenuated with time. JUNG is farther away from the epicenter than the other stations; as shown in Figure 4d, the post-seismic displacement at JUNG differs from that at the other stations. This may be due to the relatively small co-seismic displacement at this station. This indicates that the distance from the epicenter has a notable effect on the post-seismic displacement within the Korean Peninsula.

Among the unknown parameters of the function, *a* is the amplitude associated with the estimated cumulative displacement. We estimate the post-seismic relaxation completeness using the amplitude *a*. The relaxation completeness is defined as follows:

$$\mathrm{Relaxation}\;\mathrm{completeness}\;\left(\%\right)=\frac{\mathrm{the}\;\mathrm{cumulative}\;\mathrm{displacement}\;\mathrm{over}\;\mathrm{approximately}\;9\;\mathrm{years}}{\mathrm{the}\;\mathrm{estimated}\;\mathrm{final}\;\mathrm{cumulative}\;\mathrm{displacement}}$$

The relaxation completeness with the epicentral distance for each station is summarized in Table S2 of the supplementary material. Some stations that cannot be fitted with the exponential function are excluded. The average relaxation completeness for the Korean Peninsula is approximately 89%. From the relaxation completeness, we can estimate the crustal stabilization time, which is estimated from the time elapsed to reach 95% of the relaxation completeness for each station. Accordingly, it may take approximately 5–25 years for a crustal movement to stabilize following the 2011 Tohoku-Oki earthquake. The relaxation completeness showed a low correlation with the epicentral distance. It should be noted that the relaxation completeness is not proportional to the distance from the epicenter, but may instead be associated with the crustal structure of the Korean Peninsula. Ergintav et al. [1] reported the relaxation amplitudes depend on the rheology of the crust and upper mantle, and on the local conditions around the site.

## 4. Discussion

The 2011 Tohoku-Oki earthquake caused significant crustal deformation in the Korean Peninsula. After the megathrust earthquake, the crustal movements were different from those before the 2011 Tohoku-Oki earthquake. These variations in the velocity vector on the Korean Peninsula may be due to the complex effect of the post-seismic deformation and the regional geologic structure. To further analyze the crustal movement changes before and after the earthquake, we depicted the velocity vectors in five periods.

**Figure 5:**
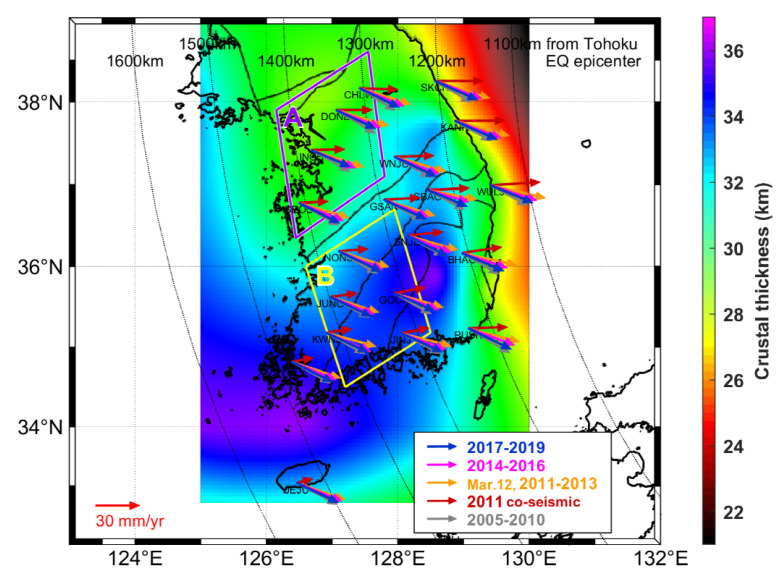
Velocity vectors of the stations and crustal thickness (from Hong et al. [29]) in South Korea in five periods. A crustal movement recovery is different according to the crustal thicknesses. The two areas ‘A’ and ‘B’ have similar epicenter distances but different crustal thicknesses. The crust thicknesses in areas ‘A’ and ‘B’, located between 1300 and 1400 km of epicentral distance, are 28–31 km and 33–36 km, respectively. The movements caused by the earthquake show a pattern of slowly returning to the pre-earthquake direction. Note: In the 2011 co-seismic stage (red arrows), the unit of magnitude is mm. It is a total displacement, not a rate.

Figure 5 shows the velocity vectors in five stages with different colors during each time period for 20 GNSS stations. We randomly selected 20 stations to clarify the figure. The Korean Peninsula moved toward the southeast in the direction of about 119 degrees during 2005–2010. The 2011 Tohoku-Oki earthquake caused a momentary yet considerable change in the entire crust of South Korea. In contrast to the long-term southeastward motion, the co-seismic movement was to the east: i.e., it changed from the southeast with the gray arrows to the east with the dark red arrows (Figure 5).

After the 2011 Tohoku-Oki earthquake, the crustal movement in the Korean Peninsula seems to be recovering to the pre-earthquake direction. However, this movement does not coincide with the direction of the pre-earthquake. During the post-seismic stage 1 (March 2012, 2011–2013) marked by orange arrows, the crust moved in the direction of 106 degrees on average from the north. This direction shows a difference of about 13 degrees compared to the pre-earthquake. The crustal movement was counterclockwise compared to the pre-earthquake motion during this period. In the next post-seismic stage (2014–2016) represented by the pink arrows, it moved in the direction of about 111 degrees on average from the north. This direction shows a difference of about 8 degrees from the pre-earthquake. Interestingly, the direction of the crustal movement is getting close to the pre-earthquake.

The direction of crustal movement from 2017 to 2019 was about 115 degrees on average from the north. This movement is marked by the blue arrows in Figure 5. During this period, the direction of crustal movement shows a difference of about 4 degrees counterclockwise from the pre-earthquake. Although there is a slight difference in the direction of the crustal movement in all GNSS stations, the crust movement of South Korea seems to be in the process of recovering in the pre-earthquake direction. As a result, we present a clear recovery pattern for the crustal movement of the Korean Peninsula during the post-seismic deformation.

The recovery patterns for the post-seismic deformation can be explained by viscoelastic relaxation, i.e., the crust deforms to return to its previous level. Tormann et al. [30] presented that the crustal stress levels quickly recovered to pre-earthquake levels within just a few years after the 2011 Tohoku-Oki earthquake. In general, when the stress is removed, the material returns to its original state. Additionally, all materials are viscoelastic under some conditions. It can be interpreted that the viscoelastic relaxation mechanism plays an important role in long-term and far-field crustal deformation [17,31]. In addition, Tanaka et al. [32] and Sun et al. [33] suggested that the post-seismic deformation be explained by viscoelastic relaxation based on the model and GPS observation, respectively. Our results also show similar characteristics with results reported by the previous studies.

The steady-state lower crust viscosity has a good correlation with crustal thickness [34]. The movement recovery in South Korea can be related to the crustal thickness. Hong et al. [19] demonstrated that the tensional stresses induced by the 2011 earthquake exceeded 6 kPa in the East Sea region, but less than 2 kPa in the Yellow Sea region. The spatial distribution of tensional strains and stresses tends to be similar to the distribution of crustal thickness in the Korean Peninsula. In order to specifically analyze the recovery pattern for the crust thickness, we divided the two areas ‘A’ and ‘B’, where are located between 1300 and 1400 km of epicentral distance, in Figure 5. The crust thicknesses in areas ‘A’ and ‘B’ are 28–31 km and 33–36 km, respectively.

**Figure 6:**
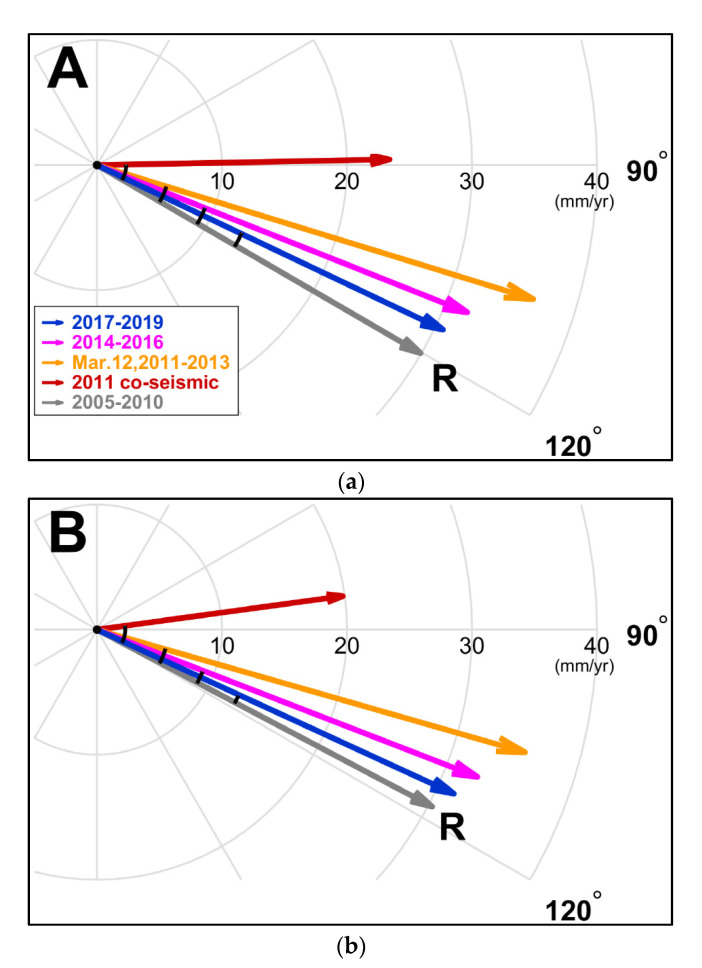
Average velocity vectors of the stations over five periods in the crustal thin (**a**) and thick (**b**) regions corresponding to the two areas ‘A’ and ‘B’ in Figure 5, respectively. The ‘R’ mark represents the pre-earthquake direction as a reference direction. The area ‘B’ with a thick crust shows a faster recovery pattern than the area ‘A’ with a thin crust. Note: In the 2011 co-seismic stage (red arrows), the unit of magnitude is mm. It is a total displacement, not a rate.

Figure 6 shows the average velocity vectors for the GNSS stations in the two areas, ‘A’ and ‘B’ in Figure 5. As previously mentioned, it is divided into five stages to calculate the average velocity vectors for the ‘A’ and ‘B’ areas. The directions for the average velocity vectors were calculated as listed in Table 2. The magnitude and direction of the velocity vector for each station over five periods are summarized in Tables S3 and S4 of the supplementary material. In the pre-seismic stage, the average directions for the crust in areas ‘A’ and ‘B’ are about 120.2 and 117.8 degrees from the north, respectively. The directions of co-seismic displacement in areas ‘A’ and ‘B’ are about 88.9 and 82.3 degrees. It means that the 2011 Tohoku earthquake dramatically changed the direction of the curst movement on the Korean Peninsula. In the post-seismic stage 1, the average directions of movements in areas ‘A’ and ‘B’ showed a difference of about 13.1 and 11.8 degrees counterclockwise from their pre-earthquake directions (‘R’) with the solid gray arrow (Figure 6). There was no significant difference between ‘A’ and ‘B’ areas in the post-seismic stage 1. However, in the post-seismic stage 2, we can see a noticeable difference between ‘A’ and ‘B’. The average direction of movement in areas ‘A’ and ‘B’ differed by about 8.5 and 6.6 degrees from the pre-quake motion (‘R’), respectively. From the results of the post-seismic stage 2, the crustal recovery in area ‘B’ seems to be faster than that in area ‘A’. The results in the post-seismic stage 3 were also very similar to those in the post-seismic stage 2. The average directions of movements in areas ‘A’ and ‘B’ showed a difference of about 4.8 and 3.1 degrees counterclockwise from their pre-earthquake directions (‘R’). Therefore, area ‘B’ with a thick crust showed a faster recovery pattern than area ‘A’ with a thin crust. The quicker recovery was more evident in the post-seismic stages 2 and 3.

Using the post-seismic relaxation completeness, we investigated the average relaxation completeness in areas ‘A’ and ‘B’, as summarized in Table 3. Additionally, the post-seismic relaxation completeness for the stations in the two areas ‘A’ and ‘B’ are summarized in Table S5 of the supplementary material. The average relaxation completeness in areas ‘A’ and ‘B’ is about 85% and 93%, respectively. The relaxation completeness of post-seismic deformation in area ‘B’, where the crust is thick, is higher than that in area ‘A’. In addition, the completeness error in area ‘B’ is also smaller than in area ‘A’. This means that the crustal movement in area ‘B’ is faster stabilizing than in area ‘A’.

Ansari and Bae [35] showed that the angular velocities of the northern and southern blocks of South Korea were different through the analysis of the block-wise rotation for each block before and after the 2011 Tohoku-Oki earthquake. They separated the northern and southern blocks by fault lines that crossed the middle of South Korea diagonally. The division by the fault lines is similar to the difference in the crustal thickness in South Korea. Therefore, different recovery patterns in areas ‘A’ and ‘B’ can be related to some crustal conditions such as crustal thickness.

In conclusion, after the 2011 Tohoku-Oki earthquake, different recovery patterns for the crustal movement of the Korean Peninsula can be directly associated with the crustal thickness. In addition, the complex structure of the Korean Peninsula’s crust may have influenced different recovery patterns.

## 5. Conclusions

In this study, we investigated decaying post-seismic deformation observed on the Korean Peninsula following the 2011 Tohoku-Oki earthquake. Using GNSS data for the 15 years from 2005 to 2019, the velocity vector of the Korean Peninsula was calculated over five periods of time. From 2005 to 2010, the crust of the Korean Peninsula moved toward the southeast in the direction of about 119 degrees with an average rate of approximately 30 mm/year. In 2011, the Tohoku-Oki earthquake caused large displacements of the crust throughout South Korea; the co-seismic offset tended to reduce with the distance from the epicenter. According to the analyzed results of changes in the magnitude and direction components of the crustal velocity vectors depending on the epicentral distance, there was a difference of a few mm/year in the magnitude between the eastern and western regions in the post-seismic stage 1 (12 March 2011 to 31 December 2013). However, in the post-seismic 3 (1 Januray 2017 to 31 December 2019), the difference in the magnitude was less than 1 mm/year between the two regions. In contrast, there was an obvious difference in the horizontal direction between the eastern and western regions before and after the 2011 earthquake. There was also a change in the average direction of the velocity vector during each time period. However, the correlation between the variation of the velocity vector and the epicentral distance decreased with time.

Additionally, we analyzed the velocity vectors for 20 GNSS stations with the crustal thickness during each time period. The crustal velocity vectors displayed different azimuths in the five periods. In the post-seismic stage, the vector direction tended to recover to the direction of the pre-earthquake. It is shown that a crustal movement recovery in the area with a thick crust is faster than in the area with a thin crust. In addition, the relaxation completeness of post-seismic deformation in the thick area is higher than in the thin area. From these results, the crustal movement recovery may be associated with the regional geologic structure (e.g., the crustal thickness). Our estimates of the decay in post-seismic deformation of the Korean Peninsula suggest that post-seismic relaxation will be complete within 5–20 years after the 2011 earthquake. We suggest that the movement characteristics of the crust in the Korean Peninsula after the earthquake are gradually recovering to their pre-earthquake motion. However, it is unclear whether the crustal movement will be recovered to the same motion as before the 2011 Tohoku-Oki earthquake after post-seismic relaxation fully completeness.

## Figures and Tables

**Table 1:** Average velocity magnitude and direction in the five periods.

Stage	Pre-Seismic Deformation	Co-seismic Deformation ^1^	Post-Seismic Stage 1	Post-Seismic Stage 2	Post-Seismic Stage 3
Period	1 January 2005 – 31 December 2010	4 March 2011 – 18 March 2011	12 March 2011 – 31 December 2013	1 January 2014 – 31 December 2016	1 January 2017 – 31 December 2019
Average magnitude (mm/year)	29.9 ± 1.3	23.2 ± 5.3	36.4 ± 1.6	32.4 ± 1.1	31.1 ± 0.9
Average direction (degree)	119.0 ± 1.4	84.4 ± 4.0	106.1 ± 1.8	110.7 ± 1.7	114.7 ± 1.7

^1^ The magnitude of co-seismic deformation has a unit of mm, as it is a total displacement, not a rate.

**Table 2:** Average velocity direction and difference by referring to the pre-seismic deformation in five periods according to the crustal thickness. The Diff. direction represents the difference angle between the average direction of each stage and the Pre-seismic (‘R’) direction (unit: degrees).

Crustal Area	Thin Area (‘A’)		Thick Area (‘B’)	
Stage	Avg. Direction	Diff. Direction	Avg. Direction	Diff. Direction
Pre-seismic (‘R’)	120.2	0.0	117.8	0.0
Co-seismic	88.9	−31.3	82.3	−35.5
Post-seismic 1	107.1	−13.1	106.0	−11.8
Post-seismic 2	111.7	−8.5	111.2	−6.6
Post-seismic 3	115.4	−4.8	114.7	−3.1

**Table 3:** Average post-seismic relaxation completeness in the two areas ‘A’ and ‘B’ in Figure 5.

Crustal Area	Thin Area (‘A’)	Thick Area (‘B’)
Average relaxation completeness (%)	85.3 ± 13.1	92.7 ± 7.0

## Data Availability

The GNSS datasets used in this article are available on GNSS DATA CENTER (http://www.gnssdata.or.kr/main/getMainView.do, accessed on 23 April 2021) from NGII and KASI.

## Supplementary Materials

The following are available online at https://www.mdpi.com/article/10.3390/s21134493/s1, Figure S1: Example of converting geocentric coordinates composed of X, Y, and Z axes to topocentric coordinates according to the north, east, and up components, Figure S2: GNSS coordinate time series of the north, east and up components for eight stations in four periods, Table S1: Velocity magnitude and direction for all stations in the five periods, Table S2: Post-seismic relaxation completeness, Table S3: Average velocity magnitude and direction for all stations in area ‘A’ with a thin crust, Table S4: Average velocity magnitude and direction for all stations in area ‘B’ with a thick crust, Table S5: Post-seismic relaxation completeness of stations in the crustal thin and thick regions.

## References

[1] Ergintav S., Burgmann R., McClusky S., Cakmak R., Reilinger R., Lenk O., Barka A., Ozener H. (2002). Postseismic deformation near the Izmit earthquake (17 August 1999, M 7.5) rupture zone. Bull. Seismol. Soc. Am..

[2] Hashimoto M., Choosakul N., Hashizume M., Takemoto S., Takiguchi H., Fukuda Y., Fujimori K. (2006). Crustal deformations associated with the great Sumatra-Andaman earthquake deduced from continuous GPS observation. Earth Planets Space.

[3] Moreno M., Rosenau M., Oncken O. (2010). 2010 Maule earthquake slip correlates with pre-seismic locking of Andean subduction zone. Nature.

[4] Ozawa S., Nishimura T., Suito H., Kobayashi T., Tobita M., Imakiire T. (2011). Coseismic and post-seismic slip of the 2011 magnitude-9 Tohoku-Oki earthquake. Nature.

[5] Hayes G.P. (2011). Rapid source characterization of the 2011 Mw 9.0 off the Pacific coast of Tohoku Earthquake. Earth Planets Space.

[6] Nettles M., Ekström G., Koss H.C. (2011). Centroid-moment-tensor analysis of the 2011 off the Pacific coast of Tohoku Earthquake and its larger foreshocks and aftershocks. Earth Planets Space.

[7] Tobita M. (2016). Combined logarithmic and exponential function model for fitting post-seismic GNSS time series after 2011 Tohoku-Oki earthquake. Earth Planets Space.

[8] Nishimura T. (2014). Pre-, co-, and post-seismic deformation of the 2011 Tohoku-Oki earthquake and its implication to a paradox in short-term and long-term deformation. J. Disaster Res..

[9] Yamagiwa S., Miyazaki S., Hirahara K., Fukahata Y. (2015). Afterslip and viscoelastic relaxation following the 2011 Tohoku-Oki earthquake (Mw9.0) inferred from inland GPS and seafloor GPS/Acoustic data. Geophys. Res. Lett..

[10] Hamdy A.M., Park P.H., Lim H.C. (2005). Horizontal deformation in South Korea from permanent GPS network data, 2000–2003. Earth Planets Space.

[11] Houng S.E., Hong T.K. (2013). Probabilistic analysis of the Korean historical earthquake records. Bull. Seismol. Soc. Am..

[12] Jin S., Park P.H. (2006). Strain accumulation in South Korea inferred from GPS measurements. Earth Planets Space.

[13] Cho J.M., Yun H.S., Lee M.R. (2011). Improvement of GPS relative positioning accuracy by using crustal deformation model in the Korean Peninsula. J. Korean Soc. Surv. Geod. Photogramm. Cartogr..

[14] Korea Meteorological Administration (2021). 2020 Seismological Annual Report.

[15] Baek J., Shin Y.H., Na S.H., Shestakov N.V., Park P.H., Cho S. (2012). Coseismic and post-seismic crustal deformations of the Korean Peninsula caused by the 2011 Mw 9.0 Tohoku earthquake, Japan, from global positioning system data. Terra Nova.

[16] Ha J., Park K.D., Won J., Heo M.B. (2014). Investigations into co-seismic deformation and strain in South Korea following the 2011 Tohoku-Oki earthquake using GPS CORS data. KSCE J. Civ. Eng..

[17] Shao Z., Zhan W., Zhang L., Xu J. (2016). Analysis of the far-field co-seismic and post-seismic responses caused by the 2011 MW 9.0 Tohoku-Oki earthquake. Pure Appl. Geophys..

[18] Zhao B., Wang W., Yang S., Peng M., Qiao X., Du R., Nie Z. (2012). Far field deformation analysis after the Mw9.0 Tohoku earthquake constrained by cGPS data. J. Seismol..

[19] Hong T.K., Lee J., Houng S.E. (2015). Long-term evolution of intraplate seismicity in stress shadows after a megathrust. Phys. Earth Planet Inter..

[20] Kim S., Ree J.H., Yoon H.S., Choi B.K., Park P.H. (2018). Crustal deformation of South Korea after the Tohoku-Oki earthquake: Deformation heterogeneity and seismic activity. Tectonics.

[21] Kim D., Park K.D., Ha J., Sohn D.H., Won J. (2016). Geodetic analysis of post-seismic crustal deformations occurring in South Korea due to the Tohoku-Oki earthquake. KSCE J. Civ. Eng..

[22] Dach R., Lutz S., Walser P., Fridez P. (2015). Bernese GNSS Software Version 5.2. User Manual.

[23] Blewitt G., Lavallée D. (2002). Effect of annual signals on geodetic velocity. J. Geophys. Res. Solid Earth.

[24] Draper N.R., Smith H. (1998). Applied Regression Analysis.

[25] Wang M., Li Q., Wang F., Zhang R., Wang Y.Z., Shi H.B., Zhang P.Z., Shen Z.K. (2011). Far-field co-seismic displacements associated with the 2011 Tohoku-oki earthquake in Japan observed by Global Positioning System. Chin. Sci. Bull..

[26] Shestakov N.V., Takahashi H., Ohzono M., Prytkov A.S., Bykov V.G., Gerasimenko M.D., Luneva M.N., Gerasimov G.N., Kolomiets A.G., Bormotov V.A. (2012). Analysis of the far-field crustal displacements caused by the 2011 Great Tohoku earthquake inferred from continuous GPS observations. Tectonophysics.

[27] Savage J.C., Prescott W.H. (1978). Asthenosphere readjustment and the earthquake cycle. J. Geophys. Res. Solid Earth.

[28] Kreemer C., Blewitt G., Maerten F. (2006). Co- and post-seismic deformation of the 28 March 2005 Nias Mw 8.7 earthquake from continuous GPS data. Geophys. Res. Lett..

[29] Hong T.K., Park S., Houng S.E. (2016). Seismotectonic properties and zonation of the far-eastern Eurasian plate around the Korean peninsula. Pure Appl. Geophys..

[30] Tormann T., Enescu B., Woessner J., Wiemer S. (2015). Randomness of megathrust earthquakes implied by rapid stress recovery after the Japan earthquake. Nature Geosci..

[31] Suito H. (2017). Importance of rheological heterogeneity for interpreting viscoelastic relaxation caused by the 2011 Tohoku-Oki earthquake. Earth Planets Space.

[32] Tanaka Y., Okuno J., Okubo S. (2007). A new method for the computation of global viscoelastic post-seismic deformation in a realistic earth model (II)-horizontal displacement. Geophys. J. Int..

[33] Sun T., Wang K., Iinuma T., Hino R., He J., Fujimoto H., Kido M., Osada Y., Miura S., Ohta Y. (2014). Prevalence of viscoelastic relaxation after the 2011 Tohoku-oki earthquake. Nature.

[34] Pollitz F.F. (2019). Lithosphere and shallow asthenosphere rheology from observations of post-earthquake relaxation. Phys. Earth Planet. Inter..

[35] Ansari K., Bae T.S. (2020). Contemporary deformation and strain analysis in South Korea based on long-term (2000–2018) GNSS measurements. Int. J. Earth Sci..

