# Supplementary material for: Decaying Post-Seismic Deformation Observed on the Korean Peninsula Following the 2011 Tohoku-Oki Earthquake
Source: Sensors (Basel). 2021 Jun 30;21(13):4493. doi: 10.3390/s21134493 (PMC8271430; doi:10.3390/s21134493)
Supplement: Supplementary file 1 [file sensors-21-04493-s001.zip › sensors-1224046-supplementary.pdf]

sensors

MDPI

Supplementary Information

Decaying post-seismic deformation observed on the Korean Peninsula following the 2011 Tohoku-Oki earthquake

Dong-Hyo Sohn [1,*], Byung-Kyu Choi [1], Sungshil Kim [2], Sun-Cheon Park [3], Won-Jin Lee [4] and Pil-Ho Park [1]

1 Space Science Division, Korea Astronomy and Space Science Institute, 776, Daedeokdae-ro, Yuseong-gu, Daejeon 34055, Korea; bkchoi@kasi.re.kr (B.-K.C.); phpark@kasi.re.kr (P.-H.P.)
2 Department of Earth Science Education, Chonnam National University, 77, Yongbong-ro, Buk-gu, Gwangju 61186, Korea; neogeo94@jnu.ac.kr
3 Earthquake and Volcano Research Division, Korea Meteorological Administration, 61, Yeouidaebang-ro 16-gil, Dongjak-gu, Seoul 07062, Korea; suncheon@kma.go.kr
4 Environmental Satellite Center, National Institute of Environmental Research, 42, Hwangyong-ro, Seo-gu, Incheon 22689, Korea; wjleeleo@korea.kr
* Correspondence: dhsohn@kasi.re.kr; Tel.: +82-42-869-5921

Figure S1. Example of converting geocentric coordinates composed of X, Y, and Z axes (left) to topocentric coordinates according to the north, east, and up components (right).

Figure S2. GNSS coordinate time series of the north, east and up components for (a) DOND, (b) JAHG, (c) JINJ, (d) JUNG, (e) KANR, (f) SEOS, (g) SNJU, (h) WULJ stations in four periods; i.e., pre-seismic (1 Jan. 2005 to 31 Dec. 2010), post-seismic stage 1 (12 Mar. 2011 to 31 Dec. 2013), post-seismic stage 2 (1 Jan. 2014 to 31 Dec. 2016), post-seismic stage 3 (1 Jan. 2017 to 31 Dec. 2019). The co-seismic (4 Mar. 2011 to 18 Mar. 2011) period is not depicted in this figure. The solid black lines (slope) are the best linear fits to the data. And the 'slope' values are the annual velocity for each component.

Table S1. Velocity magnitude and direction for all stations in the five periods. The 'Area' column represents the distinction of the stations in the two regions, the crustal thin ('A') and thick ('B'), with different crustal thicknesses in Figure 5. The last two rows, 'Mean' and 'STD', represent the average value and standard deviation, respectively.

Table S2. Post-seismic relaxation completeness. The relaxation completeness is defined as the ratio of the cumulative post-seismic displacement for approximately 9 years to the final estimated cumulative displacement. The time taken for crustal movement to stabilize is estimated from the time elapsed to reach 95% of the relaxation completeness. The 'Area' column represents the distinction of the stations in the two regions, the crustal thin ('A') and thick ('B'), with different crustal thicknesses in Figure 5.

Table S3. Average velocity magnitude and direction for all stations in area 'A' with a thin crust from Table S1.

Table S4. Average velocity magnitude and direction for all stations in area 'B' with a thick crust from Table S1.

Table S5. Post-seismic relaxation completeness of stations in the crustal thin ('A') and thick ('B') regions from Table S2 corresponding to the two areas 'A' and 'B' in Figure 5.

Citation: Sohn, D.-H.; Choi, B.-K.; Kim, S.; Park, S.-C.; Lee, W.-J.; Park, P.-H. Decaying post-seismic deformation observed on the Korean Peninsula following the 2011 Tohoku-Oki earthquake. *Sensors* **2021**, *21*, 4493. https://doi.org/10.3390/s21134493

Academic Editor: Chris Rizos

Received: 30 April 2021
Accepted: 29 June 2021
Published: 30 June 2021

Publisher's Note: MDPI stays neutral with regard to jurisdictional claims in published maps and institutional affiliations.

Copyright: © 2021 by the author. Licensee MDPI, Basel, Switzerland. This article is an open access article distributed under the terms and conditions of the Creative Commons Attribution (CC BY) license (http://creativecommons.org/licenses/by/4.0/).

Figure S1. Example of converting geocentric coordinates composed of X, Y, and Z axes (left) to topocentric coordinates according to the north, east, and up components (right).

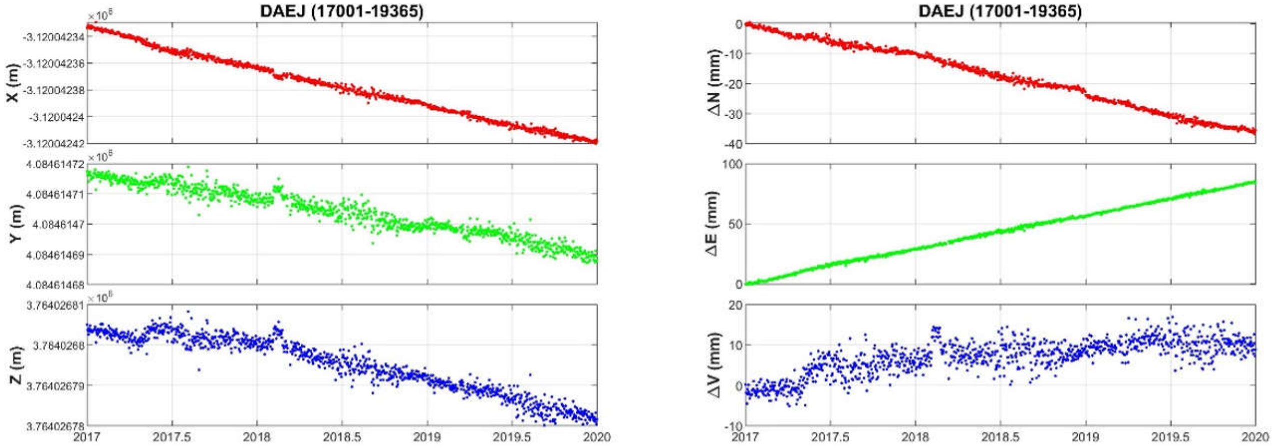

Figure S2. GNSS coordinate time series of the north, east and up components for (a) DOND, (b) JAHG, (c) JINJ, (d) JUNG, (e) KANR, (f) SEOS, (g) SNJU, (h) WULJ stations in four periods; i.e., pre-seismic (1 Jan. 2005 to 31 Dec. 2010), post-seismic stage 1 (12 Mar. 2011 to 31 Dec. 2013), post-seismic stage 2 (1 Jan. 2014 to 31 Dec. 2016), post-seismic stage 3 (1 Jan. 2017 to 31 Dec. 2019). The co-seismic (4 Mar. 2011 to 18 Mar. 2011) period is not depicted in this figure. The solid black lines (slope) are the best linear fits to the data. And the 'slope' values are the annual velocity for each component.

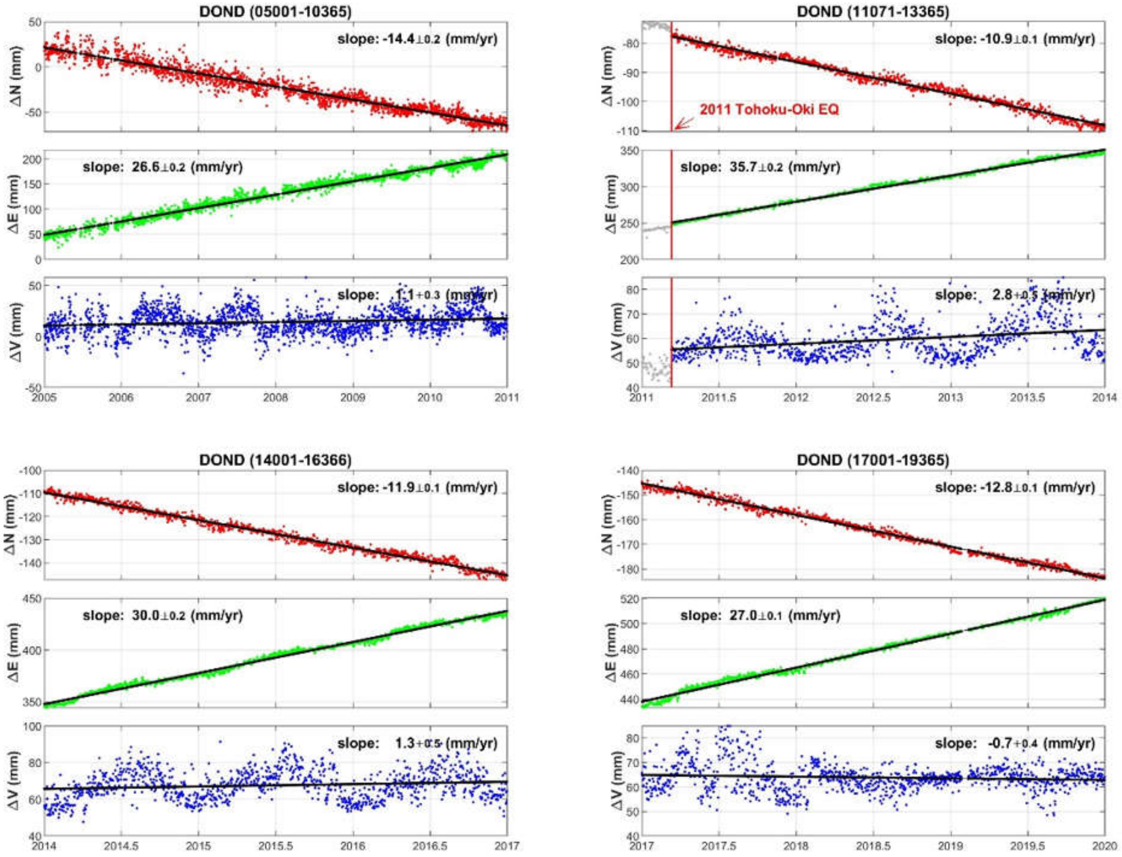

(a) DOND

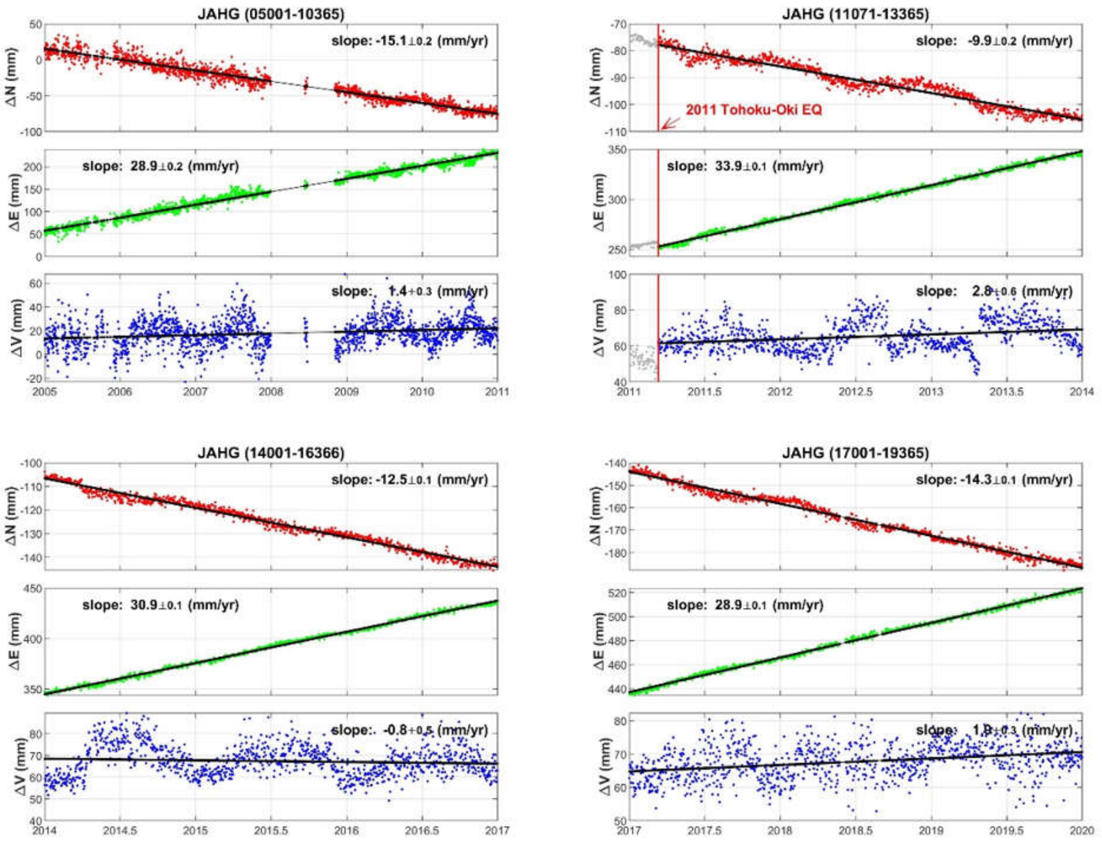
JAHG (05001-10365)
slope: -15.1±0.2 (mm/yr)
slope: 28.9±0.2 (mm/yr)
slope: 1.4±0.3 (mm/yr)
JAHG (11071-13365)
slope: -9.9±0.2 (mm/yr)
2011 Tohoku-Oki EQ
slope: 33.9±0.1 (mm/yr)
slope: 2.8±0.6 (mm/yr)
JAHG (14001-16366)
slope: -12.5±0.1 (mm/yr)
slope: 30.9±0.1 (mm/yr)
slope: -0.8±0.5 (mm/yr)
JAHG (17001-19365)
slope: -14.3±0.1 (mm/yr)
slope: 28.9±0.1 (mm/yr)
slope: 1.9±0.3 (mm/yr)
ΔN (mm)
ΔE (mm)
ΔV (mm)

(b) JAHG

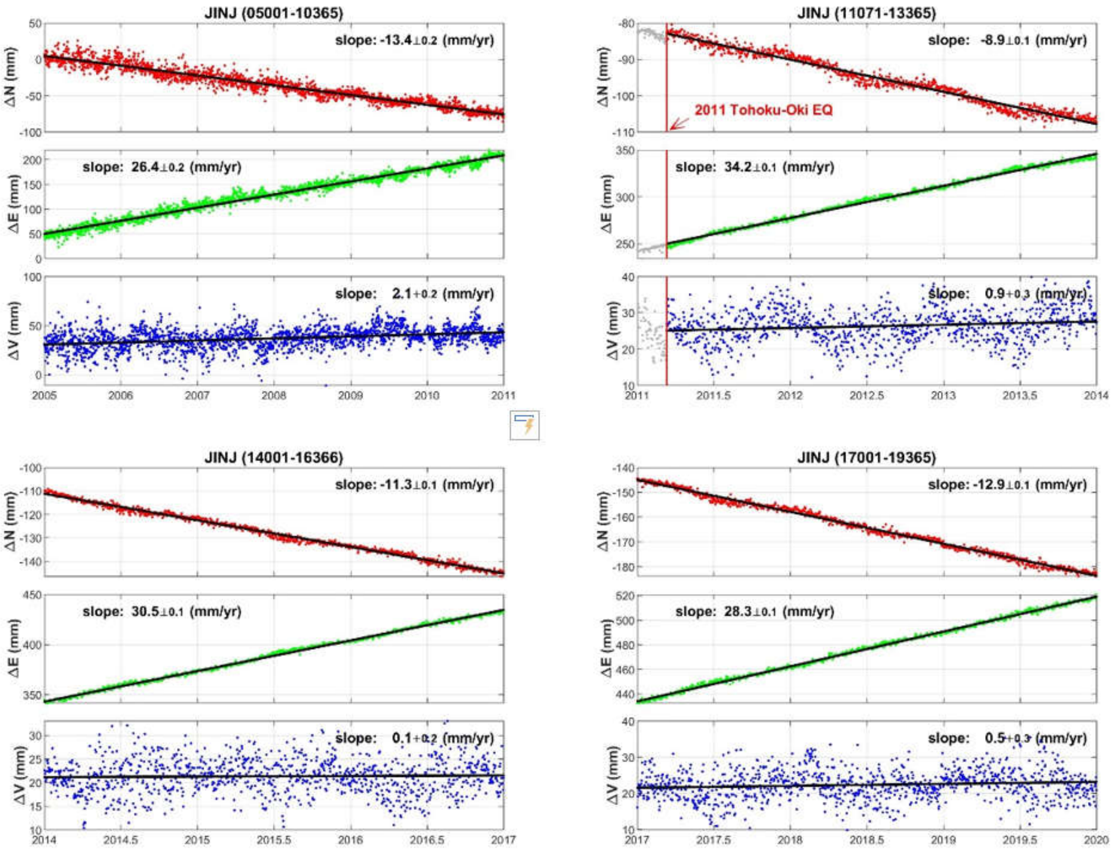
JINJ (05001-10365)
slope: -13.4±0.2 (mm/yr)
slope: 26.4±0.2 (mm/yr)
slope: 2.1±0.2 (mm/yr)
JINJ (11071-13365)
slope: -8.9±0.1 (mm/yr)
2011 Tohoku-Oki EQ
slope: 34.2±0.1 (mm/yr)
slope: 0.9±0.3 (mm/yr)
JINJ (14001-16366)
slope: -11.3±0.1 (mm/yr)
slope: 30.5±0.1 (mm/yr)
slope: 0.1±0.2 (mm/yr)
JINJ (17001-19365)
slope: -12.9±0.1 (mm/yr)
slope: 28.3±0.1 (mm/yr)
slope: 0.5±0.3 (mm/yr)
ΔN (mm)
ΔE (mm)
ΔV (mm)

(c) JINJ

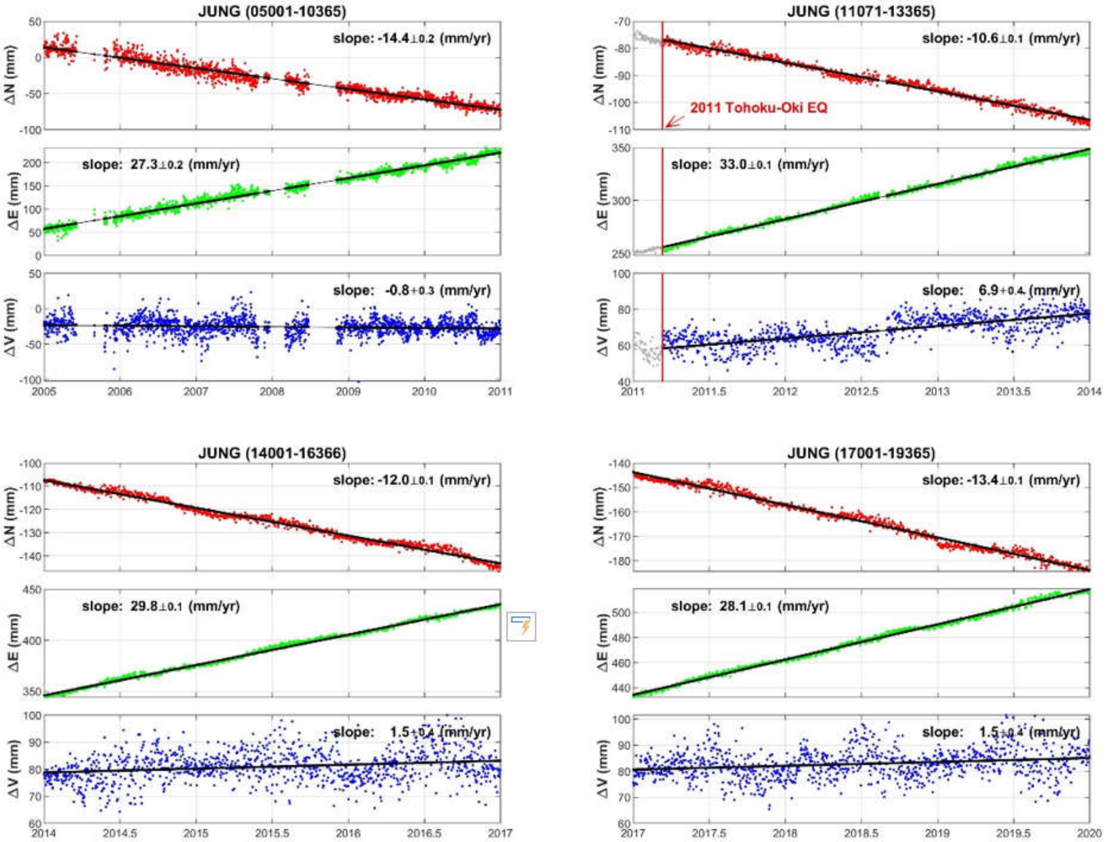
JUNG (05001-10365)
slope: -14.4±0.2 (mm/yr)
slope: 27.3±0.2 (mm/yr)
slope: -0.8+0.3 (mm/yr)
JUNG (11071-13365)
slope: -10.6±0.1 (mm/yr)
2011 Tohoku-Oki EQ
slope: 33.0±0.1 (mm/yr)
slope: 6.9+0.4 (mm/yr)
JUNG (14001-16366)
slope: -12.0±0.1 (mm/yr)
slope: 29.8±0.1 (mm/yr)
slope: 1.5+0.4 (mm/yr)
JUNG (17001-19365)
slope: -13.4±0.1 (mm/yr)
slope: 28.1±0.1 (mm/yr)
slope: 1.5±0.4 (mm/yr)
ΔN (mm)
ΔE (mm)
ΔV (mm)

(d) JUNG

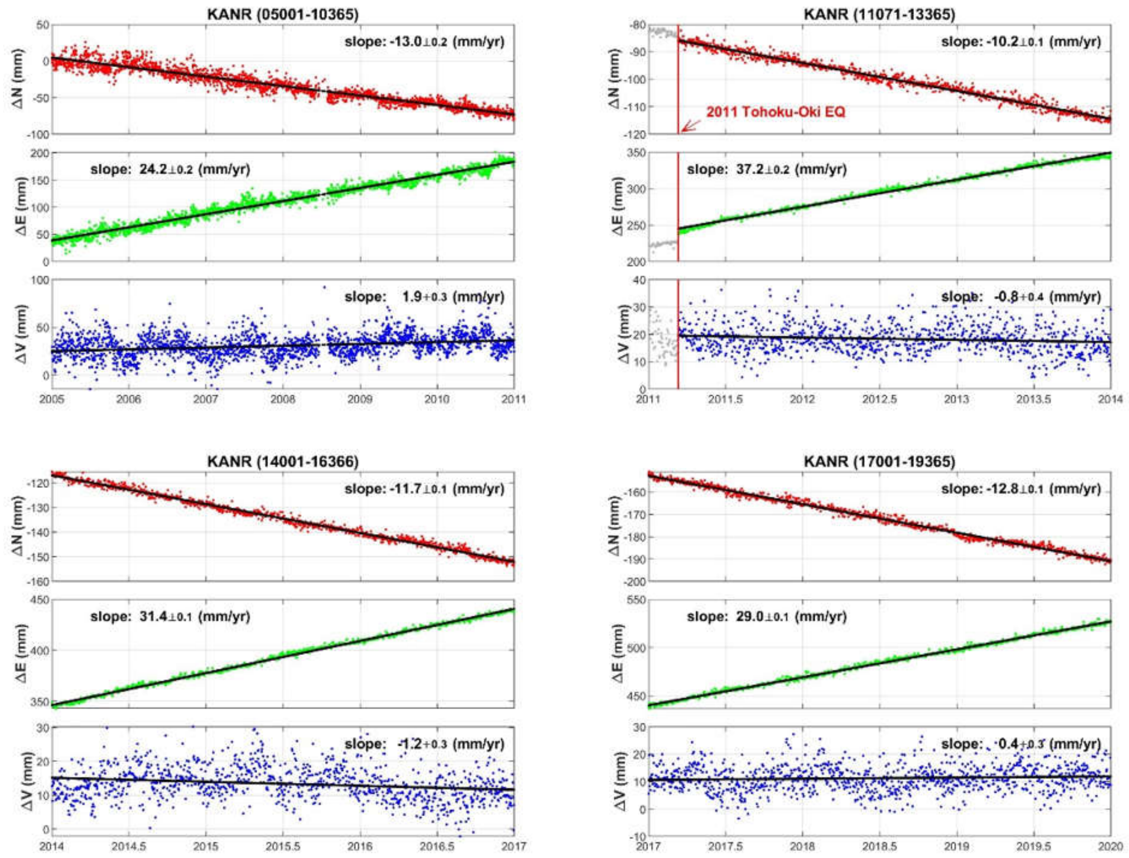
KANR (05001-10365)
slope: -13.0±0.2 (mm/yr)
slope: 24.2±0.2 (mm/yr)
slope: 1.9±0.3 (mm/yr)
KANR (11071-13365)
slope: -10.2±0.1 (mm/yr)
2011 Tohoku-Oki EQ
slope: 37.2±0.2 (mm/yr)
slope: -0.8±0.4 (mm/yr)
KANR (14001-16366)
slope: -11.7±0.1 (mm/yr)
slope: 31.4±0.1 (mm/yr)
slope: -1.2±0.3 (mm/yr)
KANR (17001-19365)
slope: -12.8±0.1 (mm/yr)
slope: 29.0±0.1 (mm/yr)
slope: 0.4±0.3 (mm/yr)
ΔN (mm)
ΔE (mm)
ΔV (mm)

(e) KANR

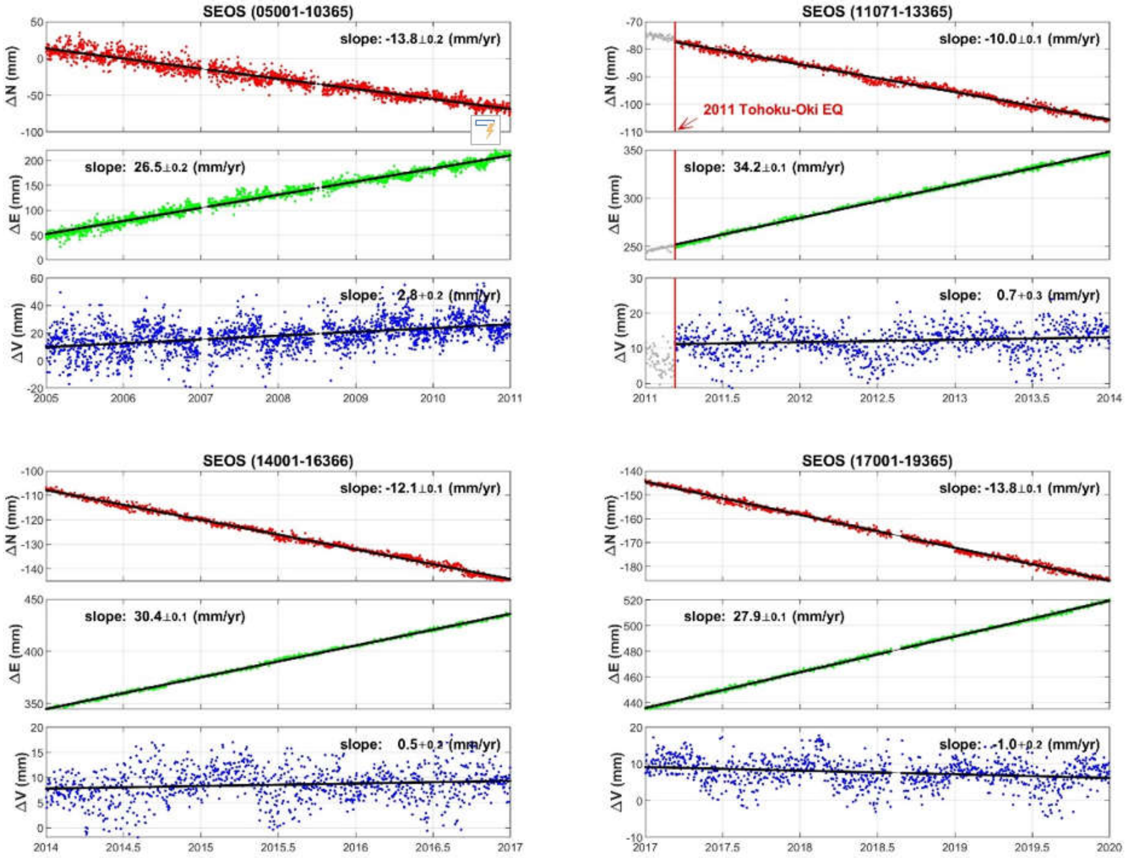
SEOS (05001-10365)
slope: -13.8±0.2 (mm/yr)
slope: 26.5±0.2 (mm/yr)
slope: 2.8±0.2 (mm/yr)
SEOS (11071-13365)
slope: -10.0±0.1 (mm/yr)
2011 Tohoku-Oki EQ
slope: 34.2±0.1 (mm/yr)
slope: 0.7±0.3 (mm/yr)
SEOS (14001-16366)
slope: -12.1±0.1 (mm/yr)
slope: 30.4±0.1 (mm/yr)
slope: 0.5±0.2 (mm/yr)
SEOS (17001-19365)
slope: -13.8±0.1 (mm/yr)
slope: 27.9±0.1 (mm/yr)
slope: -1.0±0.2 (mm/yr)
ΔN (mm)
ΔE (mm)
ΔV (mm)

(f) SEOS

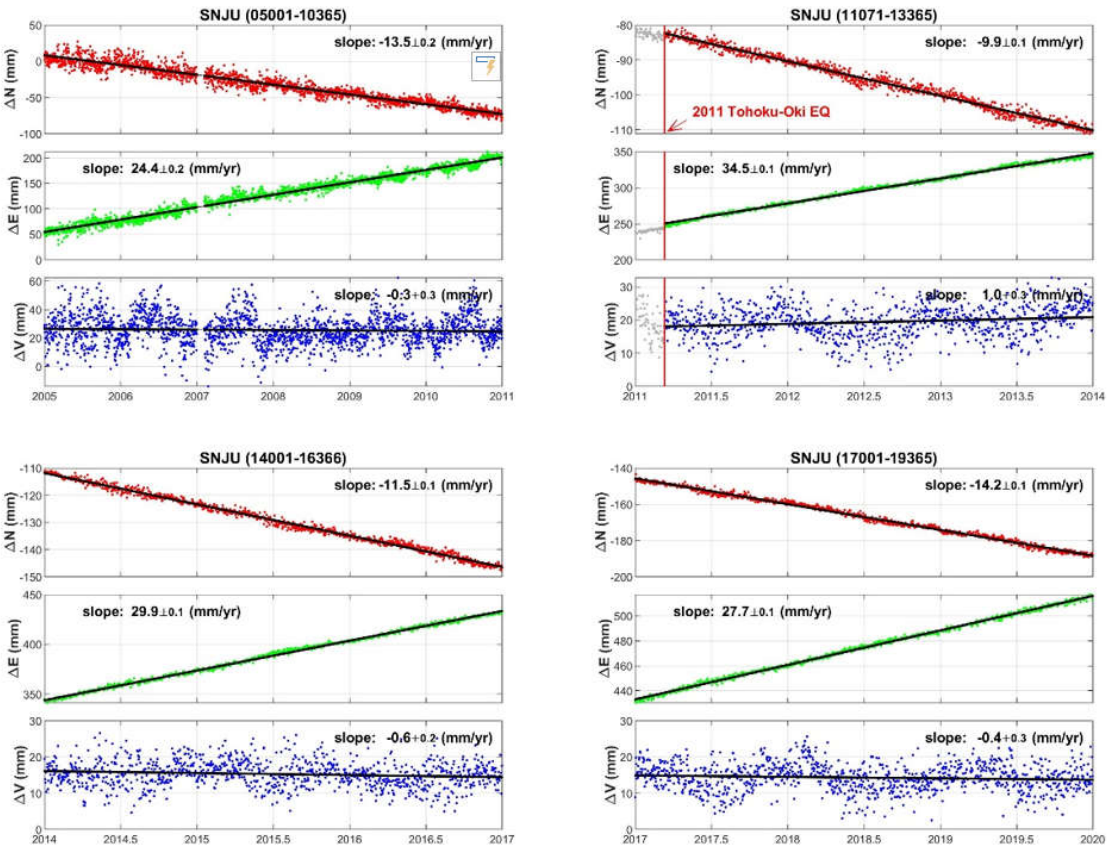
SNJU (05001-10365)
slope: -13.5±0.2 (mm/yr)
slope: 24.4±0.2 (mm/yr)
slope: -0.3±0.3 (mm/yr)
SNJU (11071-13365)
slope: -9.9±0.1 (mm/yr)
2011 Tohoku-Oki EQ
slope: 34.5±0.1 (mm/yr)
slope: 1.0±0.3 (mm/yr)
SNJU (14001-16366)
slope: -11.5±0.1 (mm/yr)
slope: 29.9±0.1 (mm/yr)
slope: -0.6±0.2 (mm/yr)
SNJU (17001-19365)
slope: -14.2±0.1 (mm/yr)
slope: 27.7±0.1 (mm/yr)
slope: -0.4±0.3 (mm/yr)
ΔN (mm)
ΔE (mm)
ΔV (mm)

(g) SNJU

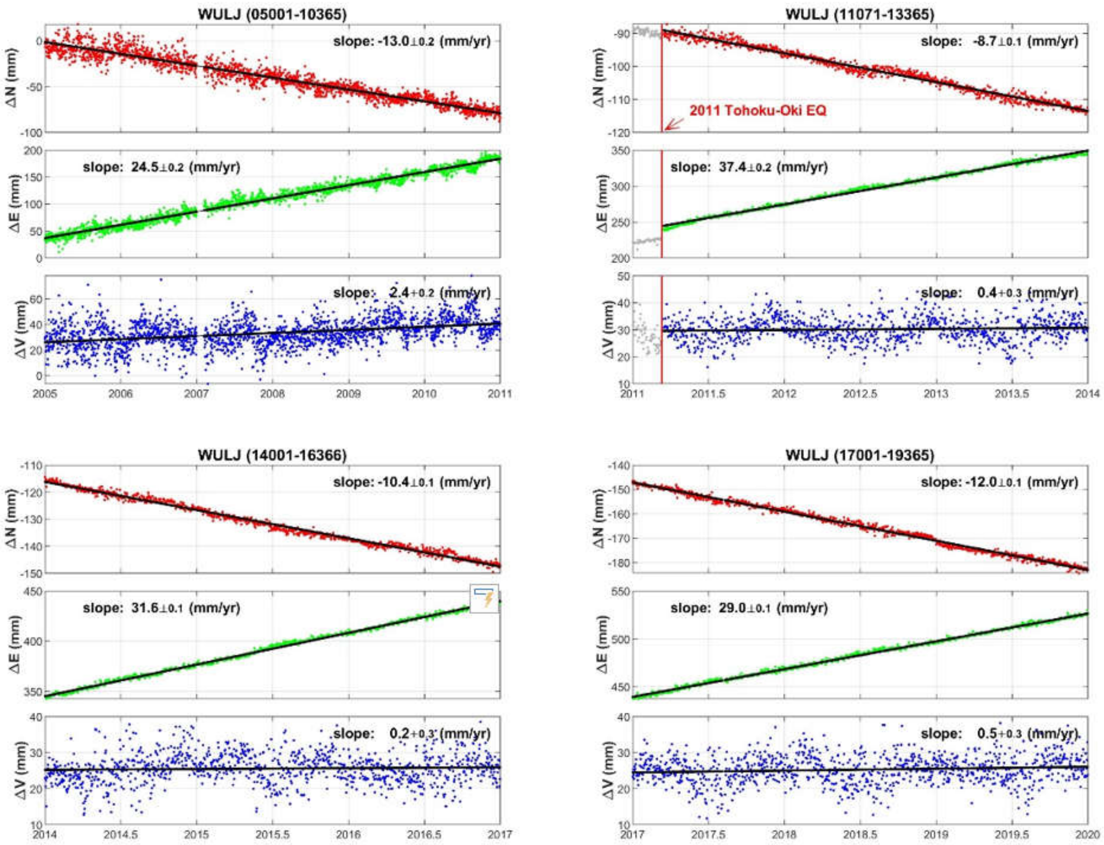

(h) WULJ

Table S1. Velocity magnitude and direction for all stations in the five periods. The 'Area' column represents the distinction of the stations in the two regions, the crustal thin ('A') and thick ('B'), with different crustal thicknesses in Figure 5. The last two rows, 'Mean' and 'STD', represent the average value and standard deviation, respectively.

	Pre-seismic		Co-seismic		Post-seismic 1		Post-seismic 2		Post-seismic 3		
SITE	Mag. (mm/yr)	Dir. (degrees)	Mag. (mm)	Dir. (degrees)	Mag. (mm/yr)	Dir. (degrees)	Mag. (mm/yr)	Dir. (degrees)	Mag. (mm/yr)	Dir. (degrees)	Area
BHAO	29.1	119.5	27.6	81.5	38.3	102.5	32.4	108.1	31.2	113.2	
CHJU	33.5	123.2	12.1	77.6	34.2	107.4	32.6	114.1	32.1	117.9	
CHLW	28.9	122.0	26.5	92.1	36.7	107.5	32.8	112.1	30.5	115.9	A
CHNG	29.2	116.2	23.5	81.9	38.2	104.4	32.1	109.4	31.3	114.3	
CHYG	31.4	118.4	19.0	85.1	35.5	107.3	32.6	111.9	31.1	115.4	
CNJU	30.0	119.1	22.9	84.6	37.2	109.5	32.8	110.3	29.7	119.0	
DAEJ	29.2	118.0	21.8	83.1	35.6	106.4	32.9	112.0	30.7	113.7	
DOND	31.2	120.9	24.6	89.9	37.3	106.9	32.3	111.6	29.9	115.4	A
GOCH	29.1	117.1	22.0	79.6	34.9	106.0	32.4	110.2	30.5	113.4	B
GSAN	29.6	117.8	25.0	86.9	36.6	107.4	32.7	111.0	31.8	114.6	
HADG	33.0	117.6	18.9	80.8	36.4	106.3	32.9	110.4	32.0	114.0	B
INCH	29.9	119.5	22.7	88.7	37.5	108.0	32.5	112.2	28.9	113.5	A
INJE	28.3	117.5	31.3	90.7	41.2	108.0	33.7	110.9	31.3	115.3	
JAHG	30.4	118.7	16.3	81.5	35.3	106.3	33.3	112.0	32.2	116.3	

JEJU	31.5	117.9	11.7	75.6	34.8	108.9	33.3	112.0	31.4	115.3	
JINJ	30.4	117.5	19.6	79.7	35.3	104.5	32.5	110.4	31.1	114.5	B
JUNG	30.3	117.7	18.6	82.3	34.7	107.7	32.1	111.9	31.1	115.5	B
JUNJ	30.7	118.3	19.5	84.2	36.6	104.7	32.7	112.3	31.8	116.4	B
KANR	28.3	118.8	34.2	89.6	38.5	105.3	33.5	110.4	31.7	113.8	
KUNW	29.2	120.4	26.9	82.0	38.6	103.7	32.2	108.3	29.9	112.1	
KWNJ	30.0	118.6	17.8	81.4	35.2	105.5	33.3	112.9	31.7	115.0	B
MKPO	30.2	118.7	15.4	79.9	35.3	108.5	32.6	112.2	31.5	116.2	
MLYN	30.2	118.8	23.8	80.6	36.5	104.8	32.4	109.5	31.4	113.0	
MUJU	28.3	117.4	22.3	83.8	36.1	106.0	32.8	108.8	31.0	114.1	B
NAMW	30.5	117.3	19.5	82.9	35.3	105.8	32.4	110.9	31.0	113.7	B
NONS	30.5	118.9	20.4	85.7	35.9	107.4	32.4	112.7	32.5	115.5	B
PAJU	28.2	120.4	23.0	89.3	36.2	105.5	27.8	110.4	31.1	114.9	A
PUSN	32.9	120.9	26.8	88.4	36.1	104.9	33.5	110.2	32.6	116.1	
SBAO	28.4	118.1	28.4	87.4	34.2	100.9	28.9	106.3	29.1	112.5	
SEOS	31.1	119.5	20.0	87.1	35.6	106.4	32.7	111.8	31.1	116.3	A
SKCH	28.9	120.2	33.1	90.2	39.3	107.6	33.9	111.0	32.1	114.1	
SKMA	30.7	120.0	23.5	87.1	36.0	108.4	32.3	112.5	31.3	116.0	A
SNJU	28.7	119.5	24.7	84.3	35.9	106.0	32.1	111.1	31.1	117.2	
SONC	30.8	119.3	18.0	80.3	32.5	104.9	31.7	105.8	30.8	111.0	
SUWN	29.5	118.9	23.6	88.2	36.1	106.8	32.6	111.3	31.8	116.0	A
TEGN	29.2	118.0	25.1	83.2	36.1	103.6	30.1	109.9	31.2	113.6	
WNJU	28.8	119.0	27.2	88.2	36.7	105.8	32.7	111.1	31.7	115.1	
WULJ	28.7	118.4	33.9	86.3	38.4	103.2	33.3	108.3	31.4	112.4	
YECH	28.6	119.3	27.1	84.8	36.5	105.8	32.3	109.9	29.3	111.7	
YOWL	28.1	121.0	27.8	78.9	37.2	105.7	32.8	110.3	31.8	114.7	
Mean	29.9	119.0	23.2	84.4	36.4	106.1	32.4	110.7	31.1	114.7	
STD	1.3	1.4	5.3	4.0	1.6	1.8	1.1	1.7	0.9	1.7	

Table S2. Post-seismic relaxation completeness. The relaxation completeness is defined as the ratio of the cumulative post-seismic displacement for approximately 9 years to the final estimated cumulative displacement. The time taken for crustal movement to stabilize is estimated from the time elapsed to reach 95% of the relaxation completeness. The 'Area' column represents the distinction of the stations in the two regions, the crustal thin ('A') and thick ('B'), with different crustal thicknesses in Figure 5.

Station ID	Epicentral distance (km)	Post-seismic displacement to 2019 (mm)	Estimated cumulative displacement (mm)	Relaxation completeness (%)	Estimated time elapsed to reach 95% of the relaxation completeness (years)	Area
WULJ	1149.1	46.4	56.8	81.7	16.3	
KANR	1182.9	48.7	58.8	82.8	15.6	
YOWL	1228.1	40.1	51.6	77.9	18.8	
TEGN	1231.1	24.4	26.1	93.5	9.8	
KUNW	1241.0	32.0	35.4	90.3	11.3	
YECH	1241.1	30.1	32.5	92.5	10.4	
WNJU	1270.1	28.5	29.2	97.3	7.2	
CHNG	1271.4	23.2	24.6	94.2	9.2	
SNJU	1274.3	25.2	31.2	80.7	17.5	
GSAN	1294.6	25.8	26.3	98.1	6.5	
CHLW	1304.9	35.6	44.7	79.7	17.6	A
GOCH	1312.9	14.9	15.0	99.6	4.6	B
JINJ	1321.1	20.2	23.7	85.3	14.7	B
MUJU	1326.9	24.8	31.3	79.3	17.3	B
CNJU	1327.4	29.0	39.7	73.1	21.8	
DOND	1338.8	29.6	30.7	96.4	7.9	A
HADG	1350.6	24.2	25.8	93.8	9.5	B
NAMW	1368.3	18.1	19.2	94.1	9.4	B
NONS	1370.4	20.0	21.5	92.9	8.3	B
JUNJ	1377.3	20.6	21.0	98.3	6.4	B
INCH	1378.7	25.5	26.6	95.6	8.5	A
CHYG	1389.1	20.5	21.9	93.7	9.6	
JUNG	1398.2	13.1	13.4	98.2	6.3	B
SEOS	1408.2	24.5	35.3	69.3	24.9	A
JAHG	1438.6	12.8	13.2	97.1	7.3	
Mean	1311.8	26.3	30.2	89.4	11.9	

Table S3. Average velocity magnitude and direction for all stations in area 'A' with a thin crust from Table S1.

	Pre-seismic		Co-seismic		Post-seismic 1		Post-seismic 2		Post-seismic 3	
station	Mag. (mm/yr)	Dir. (degrees)	Mag. (mm)	Dir. (degrees)	Mag. (mm/yr)	Dir. (degrees)	Mag. (mm/yr)	Dir. (degrees)	Mag. (mm/yr)	Dir. (degrees)
CHLW	28.9	122.0	26.5	92.1	36.7	107.5	32.8	112.1	30.5	115.9
DOND	31.2	120.9	24.6	89.9	37.3	106.9	32.3	111.6	29.9	115.4
INCH	29.9	119.5	22.7	88.7	37.5	108.0	32.5	112.2	28.9	113.5
PAJU	28.2	120.4	23.0	89.3	36.2	105.5	27.8	110.4	31.1	114.9
SEOS	31.1	119.5	20.0	87.1	35.6	106.4	32.7	111.8	31.1	116.3
SKMA	30.7	120.0	23.5	87.1	36.0	108.4	32.3	112.5	31.3	116.0
SUWN	29.5	118.9	23.6	88.2	36.1	106.8	32.6	111.3	31.8	116.0
Mean	29.9	120.2	23.4	88.9	36.5	107.1	31.9	111.7	30.7	115.4

Table S4. Average velocity magnitude and direction for all stations in area 'B' with a thick crust from Table S1.

	Pre-seismic		Co-seismic		Post-seismic 1		Post-seismic 2		Post-seismic 3	
station	Mag. (mm/yr)	Dir. (degrees)	Mag. (mm)	Dir. (degrees)	Mag. (mm/yr)	Dir. (degrees)	Mag. (mm/yr)	Dir. (degrees)	Mag. (mm/yr)	Dir. (degrees)
GOCH	29.1	117.1	22.0	79.6	34.9	106.0	32.4	110.2	30.5	113.4
HADG	33.0	117.6	18.9	80.8	36.4	106.3	32.9	110.4	32.0	114.0
JINJ	30.4	117.5	19.6	79.7	35.3	104.5	32.5	110.4	31.1	114.5
JUNG	30.3	117.7	18.6	82.3	34.7	107.7	32.1	111.9	31.1	115.5
JUNJ	30.7	118.3	19.5	84.2	36.6	104.7	32.7	112.3	31.8	116.4
KWNJ	30.0	118.6	17.8	81.4	35.2	105.5	33.3	112.9	31.7	115.0
MUJU	28.3	117.4	22.3	83.8	36.1	106.0	32.8	108.8	31.0	114.1
NAMW	30.5	117.3	19.5	82.9	35.3	105.8	32.4	110.9	31.0	113.7
NONS	30.5	118.9	20.4	85.7	35.9	107.4	32.4	112.7	32.5	115.5
Mean	30.3	117.8	19.8	82.3	35.6	106.0	32.6	111.2	31.4	114.7

Table S5. Post-seismic relaxation completeness of stations in the crustal thin ('A') and thick ('B') regions from Table S2 corresponding to the two areas 'A' and 'B' in Figure 5.

Area	Station ID	Epicentral distance (km)	Relaxation completeness (%)	Mean (%)	STD (%)
A	CHLW	1304.9	79.7	85.3	13.1
	DOND	1338.8	96.4		
	INCH	1378.7	95.6		
	SEOS	1408.2	69.3		
B	GOCH	1312.9	99.6	92.7	7.0
	JINJ	1321.1	85.3		
	MUJU	1326.9	79.3		
	HADG	1350.6	93.8		
	NAMW	1368.3	94.1		
	NONS	1370.4	92.9		
	JUNJ	1377.3	98.3		
	JUNG	1398.2	98.2		
